# Skin Grafting, Cryopreservation, and Diseases: A Review Article

**DOI:** 10.7759/cureus.30202

**Published:** 2022-10-11

**Authors:** Mohammad Adnan, Roshan K Jha, Priyanshu Verma, Harsh N Shah, Parth Singh

**Affiliations:** 1 Anatomy, Jawaharlal Nehru Medical College, Datta Meghe Institute of Medical Sciences, Wardha, IND; 2 Biochemistry, Jawaharlal Nehru Medical College, Datta Meghe Institute of Medical Sciences, Wardha, IND; 3 Biochemistry, Maharishi Markandeshwar (MM) Institute of Medical Sciences and Research, Ambala, IND; 4 Internal Medicine, Indira Gandhi Government Medical College, Nagpur, IND

**Keywords:** nutrition, skin ageing, moisturisers, stratum corneum, ultravoilet, hydrolyzed collagen

## Abstract

Food supplements may be consumed through diet and have been shown to modify skin functions, making them helpful in the management of skin aging. However, there are not many clinical trials that back up these assertions. The stratum corneum, which acts as the organism's contact with its environment, is the principal function of the epidermis of land vertebrates. Antioxidants are chemicals that slow down or prevent other molecules from oxidizing. In people's diets, their use has considerably expanded in recent years. Due to their benefits for health, nutrition, and therapy, natural antioxidants are increasingly being used in place of synthetic antioxidant components. A popular component thought to be an antioxidant is hydrolyzed collagen. With aging comes a steady loss of physiological integrity, capacity to handle stress from the inside out, and function. This is a byproduct of several intricate biological processes that are affected by diseases both local and systemic as well as constitutive and environmental variables. Systemic and constitutive (genetic) variables influence skin aging and its phenotypic manifestation. The biological process of skin aging is complicated and impacted by both external and endogenous causes. The primary contributor to skin cancer is sun exposure. Ultraviolet radiation from the sun can kill skin cells by directly absorbing DNA damage. The skin's hydration is a crucial factor that affects its mechanical and physical characteristics. This study looks at how the stratum corneum's molecular and macroscopic characteristics interact and change with skin wetness. Although there is little information written about them and even less is understood about them, moisturizers are a crucial component of a dermatologist's toolkit. There is a plethora of anticipated skin products on the market, but their true scientific function is yet unknown. These items play a well-known part in many skin problems, while occasionally being dismissed as simple cosmetics.

## Introduction and background

The skin is a crucial part of the body and serves as a defense against external environmental elements such as exposure to sunlight, extreme heat or cold, dust, and bacterial infection. Oxidative activity occurs during the metabolism of human tissues and is a natural and inevitable part of the aging process of the skin. Free radicals with one or more unpaired electrons and a reactive state are produced as a result of the oxidative process. The skin has its antioxidant defense against this oxidation process in the extracellular space, organelles, and subcellular compartments [1]. The use of donated skin from healthy homozygotic twins may help avoid these problems. Bauer published the first successful case of skin transplantation between homozygotic twins in 1927 [2]. One of the primary health problems that significantly affect many different groups of people and varies in age and intensity is burns. Despite improvements in nonsurgical and surgical burn treatments, the patient's look continues to be a public health concern. Skin transplantation is still regarded as the gold standard for surgical burn therapy. The availability of skin for grafting is one of the main challenges in burn surgery. Regarding nonsurgical treatment, a variety of skin dressings or alternatives are still an option [3].

Additionally, biologics have been used to treat kids with allergic skin conditions. Benralizumab and dupilumab are authorized for patients older than 12 years, whereas omalizumab and mepolizumab are authorized for youngsters as old as six years. Reslizumab is only permitted for patients older than 18 years. In eligible people, these identical antibodies may be introduced if asthma or reactive skin conditions are not effectively controlled [4]. The expression of genes capable of immunoregulatory function may lessen allograft rejection. Recent research suggests that viral interleukin (IL)-10 is one of the most effective ways to prevent rejection since it can lower the immune response during allotransplantation [5].

Tissue donation is protected by the Medical (Therapy, Educational, and Research) Act in Singapore. Reviewing the demographic and psychosocial characteristics that may generate hesitancy or unwillingness among healthcare providers is the goal of this study. A questionnaire-based survey with 18 items was carried out at the National Heart Centre of Singapore and the Singapore General Hospital. A total of 521 people took part in the survey. There were descriptive statistics run for the participant's demographics, the motivating elements behind tissue donation, motivating factors for discussing tissue donation, and causes for doubt or reluctance to donate tissue to a close relative. Fisher's exact test and Pearson's chi-square test were used to analyze any connections that may exist among various factors and the support for tissue donation [6].

## Review

Describe the situation

The disease known as bacteremia, or the infection of bacteria in the blood, has a high mortality rate. High rates of morbidity are linked to it. The patient's age, underlying health, and aggressiveness of the infective organism all influence the prognosis. Transfusion-transmitted infections are a rare cause of bacteremia, notwithstanding how challenging it can be to pinpoint the origin of the condition. Between one per 100,000 and one per 1,000,000 pack red blood cells or between one per 900,000 and one per 100,000 platelets are the expected incidences of bacterial spreading through donated blood. One in eight million red blood cells and one in 50,000 to 500,000 white blood cells result in fatalities. Because frozen platelets are thawed and kept at room temperature before being infused, there is a chance for any pathogens that may be present to grow before the substance is transfused, which is assumed to be the source of the greater rates of platelet transfusion. Making sure that blood used for transfusions is free of toxins is essential for further lowering infection rates. One method for accomplishing this is by meticulously preparing and washing a donor's skin at the location of the collection [7].

A systematic review and report of human skin transplant disinfection

Across the world, skin allografts are used to temporarily replace missing or damaged skin. Skin contamination that occurs naturally might also be introduced during recovery or processing. The recipients of allografts may be at risk due to this contamination. Allografts must be cultured for bacteria and disinfected, although the specific procedures and methods are not required by standards. Twelve research publications that examined the bioburden reduction techniques of skin grafts were found in a comprehensive evaluation of the literature from three databases. The most commonly mentioned disinfection technique that demonstrated lower contamination rates was the utilization of broad-range antibiotics and antifungal medicines. It was found that using 0.1% peracetic acid or 25 kGy of mid-infrared irradiation at cooler temperatures resulted in the largest decrease in skin transplant contamination rates [8].

Skin as a site of environmental stimuli exposure

Skin, the uppermost organ that protects the human body, is the surface upon which different environmental signals have the most immediate impact [9]. The number, quality, and distribution of melanin pigments produced by melanocytes determine the color of human skin, eyes, and hair, as well as how well they shield the skin from harmful ultraviolet (UV) rays and oxidative stress caused by numerous environmental pollutants. Melanocyte stem cells in the region of the follicular bulge replace melanocytes, which are located in the skin's layer of the interfollicular epidermis. Skin inflammation is brought on by a variety of stressors, including eczema, microbial infection, UV light exposure, mechanical injury, and aging [10]. Skin surface lipid (SSL) composition primarily reflects sebaceous secretion in the skin regions with the highest intensity of sebum (forehead, chest, and dorsum), which also flows from those sites to regions with lower concentrations, where the participation of cellular molecules rich in linoleic and oleic acid becomes more important [11]. Surgically removed skin from individuals who underwent a body contouring procedure was combined with discarded skin from excess belt lipectomies, breast reductions, and body lifts. After applying traction to both ends of the excised section, meshing by 3:1 plates, and covering with Vaseline gauze coated in an antiseptic solution prepared for burn covering, it can be removed by a dermatome. All patients in group III received a skin allograft from a living first-degree family (father, mother, brother, or sister), as they share about 50% of their DNA [12].

Avoiding eczema and food allergies in infants with skin care interventions

The principal goal is to evaluate the results of skin care therapies, like emollients, for the primary prevention of food allergy and eczema in babies. A secondary goal is to determine whether characteristics of study populations, such as age, inherited risks, and adherence to interventions, are connected to the most beneficial or harmful treatment outcomes for both eczema and food allergies [13].

Immune system and vitamin C

Vitamin C supports the skin's ability to scavenge free radicals and act as an infection barrier, possibly protecting against environmental oxidative stress. In phagocytic cells, such as neutrophils, an accumulation of vitamin C can encourage chemotaxis, phagocytosis, the generation of reactive oxygen species, and ultimately the death of microbes. Neutrophils eventually undergo apoptosis and are cleared by macrophages, resulting in the resolution of the inflammatory response. However, in chronic, non-healing wounds, such as those observed in diabetics, the neutrophils persist and instead undergo necrotic cell death, which can perpetuate the inflammatory response and hinder wound healing. Vitamin C's function in lymphocytes is less apparent; however, studies have indicated that it promotes B- and T-cell differentiation and proliferation, perhaps as a result of its gene-regulating properties. A lack of vitamin C lowers immunity and increases illness susceptibility [14]. The skin's distinctive form reflects the fact that its main purpose is to protect the body from the environment's irritants. The inner dermal layer, which ensures strength and suppleness, feeds the epidermis the nutrients, and also the outer epidermal layer, which is incredibly cellular and acts as a barrier, are the two layers that make up the skin. Normal skin contains high levels of vitamin C, which supports a variety of well-known and important activities, such as boosting collagen synthesis and helping the body's defense mechanisms against UV-induced photodamage. This information is occasionally used as support for introducing vitamin C to therapies; however, there is no evidence that doing so is more beneficial than just increasing dietary vitamin C intake [15].

HIV transmission by skin graft transplantation

Allograft donor selection has been affected by the worry that HIV could be transmitted through the skin of an allograft. To establish the potential presence of HIV at the period of donation, there is, however, no conclusive diagnostic test available. We examine the prevalence of HIV in human tissue, consider the potential for HIV transmission through the transplant of human allograft skin, and talk about the validity of current HIV testing to uncover solutions to enhance skin banks' HIV donor screening procedures. The risk of HIV transmission to severely burned patients could be reduced by using the polymerase chain reactions as a fast detection method for HIV, with skin biopsies in conjunction with standard regular HIV blood screening tests [16].

Banking for deceased donor skin grafts

A total of 262 dead donor skin allograft contributions were made during the past 10 years. The response revealed a considerable improvement after the community received counseling. Most of the donors were over 70 years, and most of the recruitment was done at home. In 10 years, 165 patients received tissue allografts from 249 donors. With seven deaths out of 151 recipients who had burn injuries, the outcome was good [17]. An injury to the tissue caused by electrical, thermal, chemical, cold, or radiation stress is referred to as a "burn." The skin's ability to repair and regenerate itself is hampered by deep wounds that produce dermal damage. Skin autografting is currently the gold standard of care for burn excision, but if the patient lacks donor skin or the wound is not suitable for autografting, the use of temporary bandages or skin substitutes may be absolutely necessary to hasten wound healing, lessen discomfort, avoid infection, and minimize aberrant scarring. Among the options are xenografts, cultured epithelial cells, allografts from deceased donors, and bioartificial skin replacements [18].

Delayed main burn wound closure

In the "developed" world's burn units, "early closure" in burn wounds means removing the burned tissues and replacing them within the first "five" post-burn days with graft or their substitutes. Acceptability of this method, however, may be hampered by a general lack of education and a lack of health education among the citizens in "developing" countries. A lack of dedicated and well-trained burns surgeons might make things worse. One of the growing Gulf nations in the Middle East is the Sultanate of Oman, where in November 1997, the National Burns Center at Khoula Hospital debuted "early" surgery, which quickly became a standard technique for managing burn wounds [19]. Major burn wounds that are promptly excised heal faster, are less infectious, and have a higher chance of survival. The best way to permanently heal these wounds is with the immediate application of autograft skin. However, temporary closure using a number of treatments can assist lower evaporative loss, ward off infection, alleviate discomfort, and minimize metabolic stress when donor skin harvesting is not possible or wounds are not yet suitable for autografting. The gold for such closure is fresh cadaver allograft, although alternative materials are now available, including frozen cadaver tissue, xenografts, and a number of synthetic goods. This study examines the physiology, product categories, and applications [20].

How skin substitutes are used to treat burn injuries

Large burn wounds are challenging to treat and heal. To help with this procedure, several engineered skin replacements have been created. These alternatives were created with specific goals in mind, which define the situations in which they may and should be used to enhance healing or get the burn site ready for autograft closure in the end. This article analyses some of the current skin replacements in use and explores some of the justifications for their usage. According to current viewpoints, the usage of skin substitutes is still in the early stages, and it will take some time before it is evident how they should be used in therapeutic settings [21].

Layers of skin

Each skin layer has a different width based on where in the body it is located due to differences within the thicknesses of the dermal and epidermal layers. The stratum lucidum, a second layer, is what gives the palms of the hand and the soles of the feet their thickest epidermis. Although it is thought that the upper back has the thickest dermis, histologically speaking, the upper back is regarded to just have "thin skin" since that lacks the stratum lucidum layer and has a thinner epidermis as hairless skin [22].

Split-skin grafting using allogeneic tissue in stem cell transplant recipients

**Figure 1:**
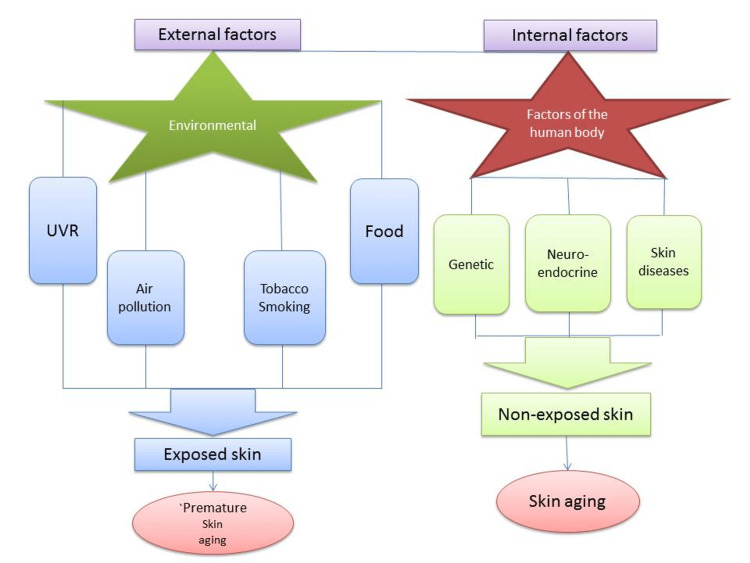
External and internal factors Adapted from: Bocheva G, Slominski RM, Slominski AT. Neuroendocrine aspects of skin aging. Int J Mol Sci. 2019;20:2798. doi: 10.3390/ijms20112798. Under Creative Common CC BY license.

We provide a rare instance of an individual who underwent satisfactory allogeneic split-thickness skin graft (STSG) transplanting and had previously undergone a bone marrow stem cell transplant. Hodgkin's bone marrow transplant (BMT) had already been done on the patient because of the myelodysplasia and non-lymphoma. Human leukocyte antigen (HLA) typing performed prior to BMT allowed for the identification of the donor and recipient, who were siblings (not twins). We achieved complete donor chimerism. Scleroderma, ichthyosis-like dryness, and severe chronic graft-versus-host disease (cGvHD) were all present in the recipient. Scalp ulceration with full thickness resulted from folliculitis. An STSG was removed under local anesthesia from the donor sister's femoral area and then transplanted into the recipient's prepared scalp ulcer without any additional anesthesia [23]. We conducted an allogeneic donor skin transplant in seven adult patients following allogeneic hematopoietic stem transplant surgery for cGvHD-associated refractory skin ulcers. Serious cGvHD-related refractory skin ulcers continue to be linked with significant morbidity and mortality. While split skin grafts (SSG) were performed on four patients, a full-thickness skin transplant was performed on one patient for two tiny, refractory ankle ulcers, and one patient got in vitro extended donor keratinocyte grafts made from the original unrelated donor's hair roots. An extensive deep fascial defect of the lower leg was first filled with an autologous larger omentum-free graft in one more patient before being filled with an allogeneic SSG (Figure 1) [24].

Storing human skin

Three skin grafting innovations led to significant improvements in the care for burn injuries. Firstly, it was discovered that the dermal layer is the most crucial component of graft in creating a new, durable, resilient surface. Secondly, it was shown that deep islands of hair follicles and sebaceous gland epithelium regrow at the donor site following the excision of a partial-thickness graft, allowing grafts to be cut thicker rather than as thin as feasible. The dermis might be transplanted without having to be as thin as feasible disrupting the areas of healing. When the grafts were thicker, it was possible to build tools for cutting bigger grafts. The split-thickness graft was the name given to these bigger grafts, and for the first in terms of square feet, it took a long time to effectively resurface big regions instead of millimeters square [25]. Skin banking was introduced in 1994 by the Melbourne-based Donor Tissue Bank of Victoria (DTBV). It is still the only skin bank in operation in Australia, processing cadaveric skin that has been cryopreserved for use in treating burns. Since the program's creation, there has been a steady rise in the demand for transplanted skin in Australia. Several major incidents or calamities, in both Australia and overseas, required the bank to provide aid. Demand is always greater than supply, thus the DTBV had to come up with measures to enhance the availability of allograft skin on a national level since there were no other local skin banks [26]. The treatment of individuals with severe burns may benefit greatly from cadaveric allograft skin. Estimating the present popularity and levels of usage of transplant skin in the US, however, is challenging. In the American Burn Association's Directory of Burn Care Resources for North America 1991-1992, which lists 140 medical directors of US burn centers and 40 skin banks, a poll of these individuals was conducted. For skin bank and burn directors, respectively, the number of responses was 45% and 38%. At the participating burn centers, 12% of patients who were hospitalized received treatment with allograft skin. Although just 47% of skin banks could provide fresh cadaver skin, 69% of burn center directors opted to utilize fresh skin. This study, which was presented to a Tissue Bank Special Interest group at the American Burns Association annual meeting in 1993, tabulated survey results as well as a review and discussion of potential future directions of replacement and skin banking research [27].

Cryopreservation's clinical effects on split-thickness allografts in the porcine model

A possible substitute for human cadaveric allografts (HCA) in the treatment of severely burned patients is pig xenografts that have undergone genetic engineering. However, if preservation and lengthy storage, without cellular viability loss, were possible, their therapeutic utility would be greatly increased. This study's goal was to determine the direct effects of cryopreservation and storage time on vital in vivo and in vitro characteristics that are required for an effective, perhaps equal replacement for HCA. In this study, viable porcine skin grafts that had been constantly frozen for more than seven years were contrasted with similarly prepared skin grafts that had been kept frozen for only 15 minutes [28]. When freshly collected allogeneic skin grafts are not available, it is thought that frozen human allogeneic skin grafts are a viable substitute. However, there is little functional and histological knowledge on how cryopreservation affects allogeneic skin transplants, particularly those that overcome mismatched histocompatibility barriers. To compare fresh and frozen skin grafts across major and minor histocompatibility barriers, we used a small-scale pig model. Our findings are relevant to the existing clinical procedures requiring allogeneic grafting and they may enable future, transient wound treatments using frozen xenografts made of genetically engineered pig skin since porcine skin and human skin share several physical and immunological characteristics [29].

Skin diseases

Peeling Skin Syndrome

The two types of peeling skin syndrome (PSS), i.e., acral PSS and generalized PSS, are uncommon autosomal recessive cutaneous genodermatoses. The general form now includes type A non-inflammatory, type B inflammatory, and type C. A single missense mutation in CHST8, the gene that codes for Golgi transmembrane N-acetylgalactosamine 4-O-sulphotransferase, results in PSS type A. As seen in our example, this mutation leads to the intracellular breakage of corneocytes, which results in asymptomatic skin peeling. Congenital ichthyosis or erythematous patches that migrate and have a peeling border are to blame for the clinical similarity between PSS type B and Netherton syndrome [30].

Chromhidrosis

Yonge described chromhidrosis for the first time in 1709. It is an uncommon disorder characterized by the discharge of colored sweat. There are three subtypes of chromhidrosis: apocrine, eccrine, and pseudochromhidrosis [31].

Necrobiosis Lipoidica

Necrobiosis lipoidica is a granuloma illness that frequently affects the lower limbs and manifests as indolent atrophic plaques. Several case studies detail various therapy options with varying degrees of effectiveness and propose potential correlations. Squamous cell carcinoma growth and ulceration are significant side effects. Despite therapy, the disease's course is frequently indolent and recurring [32].

Morgellons Disease

It is a stressful and debilitating illness to have Morgellons disease. Multiple cutaneous wounds that are not healing are a frequent presentation for patients. Patients frequently give samples to the doctor and blame the problem on protruding fibers or other things. The initial theories for the origin of this disorder ranged widely and were hotly contested, from infectious to mental [33].

Erythropoietic Protoporphyria

The final enzyme in the heme biosynthetic pathways and the cause of erythropoietic protoporphyria is ferrochelatase partial deficiency. After the first exposure to sunlight in early infancy or youth, photosensitivity develops in erythropoietic protoporphyria. There have been reports of erythropoietic protoporphyria all around the world; however, its epidemiology varies by locale. After age 10, it was discovered that 20% of the Japanese patients had erythropoietic protoporphyria symptoms [34].

Eruptive Xanthomas

Localized lipid deposits known as xanthomas are linked to lipid abnormalities and can be seen in the skin, tendons, and subcutaneous tissue. This disorder's hyperlipidemia may be brought on by a basic genetic flaw, a secondary condition, or perhaps both. Such a skin exanthem may be the initial indication of cardiovascular risk [35].

## Conclusions

A protein that is easily digested into human plasma is hydrolyzed collagen. Due to its advantages for the skin and biocompatibility, it is secure and highly sought after in the nutraceutical business. As a result of differentiation, the lipid content of the epidermis substantially changes. The final result of differentiation is a combination of ceramides, fatty acids, and cholesterol. The stratum corneum's permeability barrier is supported by these lipids. Extrinsic aging can be stopped, while natural aging is genetically determined. Aesthetic dermatology must play an important part in the prevention, regeneration, and or delaying skin aging by merging understanding of potential systemic and local therapy, instrumental gadgets, and invasive procedures, filling the gap left by the absence of technical investigations and emerging as one of the core areas of aging research. Making an effort to remove time traces from the skin will support "healthy aging" despite cosmetic methods.
